# Revisional Surgery Due to Weight Regain or Insufficient Weight Loss Following One Anastomosis Gastric Bypass

**DOI:** 10.7759/cureus.74799

**Published:** 2024-11-29

**Authors:** Adam Abu-Abeid, Adi Litmanovich, Shai M Eldar, Guy Lahat, Andrei Keidar

**Affiliations:** 1 General Surgery, Tel Aviv Sourasky Medical Center, Tel Aviv University, Tel Aviv, ISR; 2 Surgery, Tel Aviv Sourasky Medical Center, Tel Aviv University, Tel Aviv, ISR

**Keywords:** insufficient weight loss, one anastomosis gastric bypass, revisional surgery, severe obesity, weight regain

## Abstract

Introduction: One anastomosis gastric bypass (OAGB) is a common procedure associated with satisfactory outcomes. Revisional surgery due to weight regain or insufficient weight loss (WR/IWL) after OAGB is underreported.

Methods: A retrospective analysis of a single-bariatric surgeon database was conducted. All patients undergoing revisional surgery after OAGB due to WR/IWL were included.

Results: Ten patients were included in this case series. The median time between OAGB and revisional surgery was 44.9 months. The median body mass index (BMI) was 41.6 at pre-OAGB and 38.6 at pre-revision. The median age at OAGB revision was 40 years. The median biliopancreatic limb (BPL) length at revision was 160 cm. BPL elongation was performed in three patients, conversion to Roux-en-Y gastric bypass (RYGB) with BPL elongation in three patients, RYGB without BPL elongation in three patients, and one patient underwent conversion to single anastomosis duodeno-ileal bypass with sleeve (SADI-S). Pouch resizing was performed in four patients. One patient was reoperated due to a staple line leak from pouch resizing after conversion to RYGB. At a mean postoperative follow-up of 19 months, the median BMI and total weight loss were 30 and 18%, respectively. Higher weight loss outcomes occurred with BPL elongation (average: 24.8%) and SADI-S (average: 23.4%), while conversion to RYGB, with or without elongation, showed lower weight loss outcomes (11.5% and 7.8%, respectively).

Conclusions: WR/IWL after OAGB can be encountered during follow-up. In this case series, several options of revisions were evaluated, and BPL elongation or conversion to SADI-S correlated with better weight loss outcomes.

## Introduction

Severe obesity is an epidemic representing one of the most challenging public health issues and is expected to double in prevalence by 2035 [1]. Metabolic and bariatric surgery (MBS) is one of the most effective treatments for severe obesity, as it shows sustained weight loss outcomes in the long term, as well as resolution of obesity-related diseases, decreased rates of cancer, and decreased mortality [2-4].

One anastomosis gastric bypass (OAGB) is an MBS that is continuously increasing in popularity owing to its safety profile and satisfactory outcomes, both as a primary and a revisional procedure [5-7]. In fact, in the last report of the International Federation for the Surgery of Obesity and Metabolic Disorders (IFSO) regarding worldwide trends, it is now the third most commonly performed MBS worldwide and comprises 7.6% of MBS worldwide, and the highest prevalence was seen in the European and Asia Pacific Chapters (16% and 14.9%, respectively) [8].

Weight regain or insufficient weight loss (WR/IWL) after MBS is sometimes encountered during patient follow-up, and the rates can be as high as 50%, especially after restrictive procedures [9-11]. These rates are reported to be lower in procedures combining hypo-absorption [12]. Studies on the surgical revision of OAGB due to WR/IWL are lacking. In fact, and to our knowledge, there is only one study reporting the outcomes of pouch resizing after OAGB due to WR/IWL [13], and there are no studies demonstrating other surgical revision options.

The aim of this study is to describe a case series of patients undergoing various surgical revisions of OAGB due to WR/IWL.

## Materials and methods

This is a retrospective study from a prospectively maintained patient registry of a single MBS surgeon. All patients undergoing surgical revision due to WR/IWL after OAGB were included. WR/IWL was defined as a weight gain of at least 10 kg after reaching the weight nadir or a percentage of total weight loss (TWL) below 20% [14,15]. Data captured included baseline patient characteristics, including age, gender, pre-OAGB body mass index (BMI), and previous bariatric surgeries. Additional data regarding perioperative outcomes included BMI at the time of revision, type of surgical revision, perioperative complications, and length of hospital stay (LOS).

We included data regarding follow-up, such as last follow-up data, last BMI, and TWL.

The study was approved by the Institutional Review Board and was performed in accordance with the ethical standards of the institutional and/or national research committee and with the 1964 Declaration of Helsinki and its later amendments or comparable ethical standards. Informed consent does not apply to this study.

Patients

All patients underwent a multidisciplinary evaluation prior to surgery, which included evaluation by the MBS surgeon, MBS dietitian, and psychologist. All patients underwent an extensive blood panel, upper gastrointestinal (GI) series, upper GI endoscopy, and abdominal ultrasonography and were referred to additional tests as indicated by the surgeon. Indications for surgery were a BMI of at least 30 kg/m^2^.

Indications for pouch resizing

All patients undergo the aforementioned evaluation prior to revisional surgery, and all the following considerations are taken when deciding on pouch resizing such as patient history taking regarding the patient’s food intake capacity during meals, the structure and volume of the pouch are evaluated using an upper GI series with an assessment for any dilation, and lastly, intraoperatively, the pouch is inflated with fluid through a nasogastric tube to assess its size and volume.

Surgical revisions

All procedures are performed in a laparoscopic approach under general anesthesia. All surgeries are initiated by lysis of adhesions to reveal the gastric pouch and the gastro-jejunal anastomosis. The pouch is evaluated if adequate in length and volume and resized as indicated against a 34-Fr bougie. The biliopancreatic limb (BPL) length is then measured starting from the ligament of Treitz distally to the anastomosis. The common channel (CC) is then measured from the anastomosis to the ileocecal valve. The types of surgical revisions include the following.

BPL Elongation

The anastomosis is transected at the level of the previous staple line using a linear stapler, taking care not to narrow the small bowel diameter. The gastro-jejunal anastomosis is then performed distally, assuring at least 300 cm CC. Pouch resizing was performed as indicated. A routine blue dye leak test was performed.

Conversion to Roux-en-Y Gastric Bypass (RYGB) With BPL Elongation

The anastomosis was transected as noted above, and the pouch was resized as indicated. The BPL was lengthened, assuring a CC of at least 200-250 cm. The alimentary limb was measured to at least 100 cm. A routine blue dye leak test was performed.

Conversion to RYGB Without BPL Elongation

The BPL was transected from the anastomosis and was anastomosed at least 100 cm distally, assuring at least 300 cm of CC. Pouch resizing and trimming were performed as indicated. Mesenteric defects were closed routinely. A routine blue dye leak test was performed.

Conversion to Single Anastomosis Duodeno-Ileal Bypass With Sleeve (SADI-S) Gastrectomy

The gastro-jejunal anastomosis was transected as noted above. A resection of the gastric remnant was performed, and a hand-sewn gastro-gastrostomy was created. Then, the first part of the duodenum was transected after mobilization at approximately 4 cm distal to the pylorus. The CC was then measured from the ileocecal valve proximally to 250 cm, and an end-to-side hand-sewn anastomosis of the duodenum to the omega loop of the ileum was performed. A routine blue dye leak and patency test were performed for both the sleeve gastrectomy and the duodeno-ileal anastomosis.

Statistical analysis

Statistical analysis was performed using IBM SPSS Statistics for Windows, Version 27 (Released 2020; IBM Corp., Armonk, New York, United States). Continuous variables are presented as median or mean and standard deviation (SD). Proportions are presented as n (%).

## Results

Ten patients underwent surgical revision due to WR/IWL after OAGB. The time interval between OAGB and revision was 44.9 months. The incidence of surgical revision due to WR/IWL after OAGB for this surgeon is 10/1100 (0.9%). The baseline characteristics, perioperative, and follow-up data are shown in Table 1. There were eight women in the cohort (80%), and the median age at OAGB revision was 40 years. The BMI prior to OAGB was 41.6 kg/m², and prior to revisional surgery, it was 38.6 kg/m². The median BPL length at revision was 160 cm.

**Table 1:** Baseline characteristics of patients undergoing OAGB conversion due to weight regain and/or insufficient weight loss

Patient	Gender	Age (years)	BMI (kg/m^2^) before OAGB conversion	Type of revision	Complications	Length of stay* (days)	Last F/U BMI (kg/m^2^)	TWL (%)	F/U period (months)
1	F	38	35.4	BPL Elongation	-	3	27.8	21.5	20
2	F	38	39.3	Conversion to RYGB without BPL elongation	Leak from pouch resizing	2	30.1	23.5	25
3	M	45	40.8	Conversion to RYGB without BPL elongation	-	2	40.8	0	51
4	F	39	33.1	Conversion to RYGB with BPL elongation	-	3	29.4	11.1	44
5	F	45	30.0	Conversion to RYGB without BPL elongation	-	2	30.0	0	31
6	F	37	42.8	BPL Elongation	-	3	32.7	23.7	18
7	M	44	41.0	BPL Elongation	-	2	29.0	29.2	17
8	F	61	39.8	Conversion to RYGB with BPL elongation	Fluid collection	7	36.3	8.8	9
9	F	38	36.1	Conversion to RYGB with BPL elongation	-	2	30.8	14.4	8
10	F	40	37.9	Conversion to SADI-S	-	4	29.1	23.4	6
OAGB: one anastomosis gastric bypass; BMI: body mass index; RYGB: Roux-en-Y gastric bypass; BPL: biliopancreatic limb; SADI-S: single anastomosis duodeno-ileal bypass with sleeve; F/U follow-up; TWL: total weight loss. *Length of stay does not refer to later readmissions.									

Surgical revision technical data and complications are shown in Table 2. BPL elongation was performed in three patients; the indication for surgery in these patients was solely due to WR/IWL, and elongation was feasible as the CC was adequate (mean: 280 cm). RYGB with BPL elongation was performed in three patients; the indication for surgery in these patients was due to WR/IWL with symptoms suggestive of bile reflux. BPL elongation was feasible as the CC length was adequate (mean: 260 cm). RYGB without BPL elongation was performed in three patients; the indication for surgery was mainly due to bile reflux and WR/IWL. The mean CC length in this group was 455 cm. Conversion to SADI-S was performed in one patient; the indication was mainly due to WR/IWL. There was one major complication in a patient undergoing conversion RYGB requiring reoperation due to a staple line leak from pouch resizing.

**Table 2:** Operative data of patients undergoing revisional surgery due to WR/IWL after OAGB

Type of revision	Patient	Pouch resizing	Measured BPL length (cm)	TSB length (cm)	Revised BPL length (cm)	Common channel length (cm)
BPL elongation	1	Yes	130	690	370	320
	6	No	170	650	350	300
	7	Yes	170	730	500	230
Conversion to RYGB with BPL elongation	4	Yes	200	500	270	230
	8	Yes	120	450	180	270
	9	Yes	120	530	250	280
Conversion to RYGB without BPL elongation	2	Yes	110	380	Not revised	270
	3	Yes	120	700	Not revised	580
	4	Yes	180	700	Not revised	520
Conversion to SADI-S	10	No	200	800	550	250
WR/IWL: weight regain/insufficient weight loss; OAGB: one anastomosis gastric bypass; BPL: biliopancreatic limb; TSB: total small bowel; RYGB: Roux-en-Y gastric bypass; SADI-S: single anastomosis duodeno-ileal bypass with sleeve						

Follow-up

**Figure 1:**
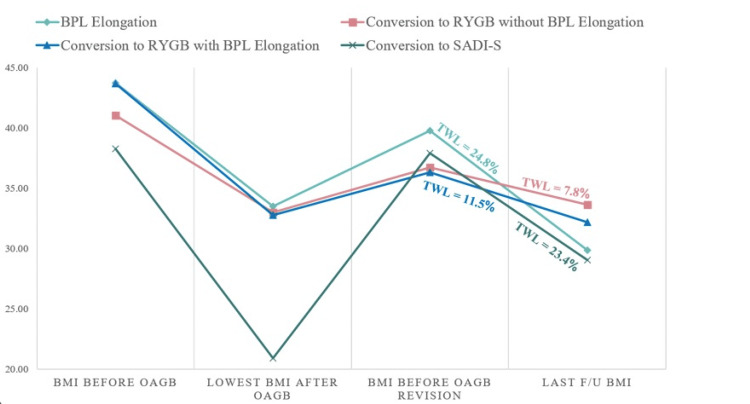
Weight trends in patients undergoing OAGB revision due to weight regain and/or insufficient weight loss It shows the BMI trends of patients prior to OAGB, nadir BMI post-OAGB, pre-revisional BMI, and BMI at the last follow-up. BPL: biliopancreatic limb; RYGB: Roux-en-Y gastric bypass; SADI-S: single anastomosis duodeno-ileal bypass with sleeve; TWL: total weight loss; OAGB: one anastomosis gastric bypass; BMI: body mass index; F/U: follow-up

At a mean postoperative follow-up of 19 months, the median BMI and TWL were 30.04 kg/m^2^ and 17.97%, respectively. The BMI trends and TWL are shown in Figure 1. A higher TWL was seen with BPL elongation (TWL 24.8%) and SADI-S (TWL 23.4%), while conversion to RYGB, with or without elongation, showed the lowest TWL (11.5% and 7.8%, respectively). All patients were compliant with follow-up and vitamin supplements. There were no patients diagnosed with protein-calorie malnutrition, and only one patient (patient number 7) had mild iron deficiency anemia.

## Discussion

Revisional surgery due to WR/IWL after OAGB is uncommon yet could be encountered during the follow-up after surgery. This case series evaluated patients undergoing revisional surgery after OAGB due to WR/IWL.

WR/IWL after MBS may be due to several reasons, which include technical dysfunction due to pouch enlargement, short BPL length, or gastro-gastric fistula, but in most cases, it is mainly due to non-compliance to dietary regulation, dysregulated eating, and physiological compensatory mechanisms leading to increased appetite [16]. WR after OAGB was reported to occur in 1-3% of patients (depending on the WR definition) in a multicenter study from India by Baig et al. [17]. Interestingly, they also reported that WR after SG and RYGB was 15.9-35.1% and 5-14.6%, respectively. This could be explained possibly due to the lower cohort of patients in the OAGB groups. It is important to emphasize that there is no consensus on the exact definition of WR/IWL after MBS, and this most probably causes variations in the different rates reported.

Revisional surgery after OAGB due to WR/IWL was reported to occur in 0.2% of patients in a retrospective analysis by Liagre et al. [18], which was significantly lower than in patients after RYGB (2.4%). In the Italian multi-institutional survey of revisional surgery after OAGB [19], the rate of reoperation due to WR was 0.48%. The rate of revisional surgery due to WR/IWL in this case series was 0.9%. This number could possibly be higher, as some patients could be operated on in other institutions. Despite this, it is close to the range of revisional surgery in the aforementioned reports.

The type of revisional surgery after OAGB is underreported. Ferro et al. [13] analyzed 23 patients with weight regain after gastric bypass (11 following OAGB and 12 following RYGB) who underwent resizing of the gastric pouch. They reported two patients with early infection complications, of which one required reoperation. In a study of pouch resizing after RYGB by Iannelli et al. [20], there were 3/20 patients with intra-abdominal abscesses. In our series, there was one patient (10%) with a staple line leak from pouch resizing. Pouch resizing is an important part of this revisional surgery, leading to increased weight loss, as it probably causes further restriction, especially in the case of dilated pouches. Nevertheless, we believe that pouch resizing should be individualized to each patient as the complication rate seems high.

Ferro et al. [13] reported a TWL of 19.6% in a mean follow-up of 24 months. In our case series, the mean follow-up time was 19 months, and the mean TWL was 18%. Better TWL outcomes were seen in procedures combining BPL elongation (TWL 24.8%) and SADI-S (TWL 23.4%). In a Metabolic and Bariatric Surgery Accreditation and Quality Improvement Program (MBSAQIP) analysis of patients undergoing BPL elongation after RYGB, the overall complication rate was comparable to RYGB; however, there was a meaningfully higher rate of reoperations in BPL elongation (3.3% vs. 1.9%) [21]. In a systematic review and meta-analyses regarding limb distalization after RYGB [22], the percentage of excess weight loss was reported to be 50.8%. In a review of the literature regarding the treatment of WR after RYGB, the percentage of excess weight loss may increase from 30% before revision to 69% at five years when performing distalization of the BPL [23]. BPL revision could possibly be an important factor in defining patient outcomes; the complication rates reported are low, and it is considered relatively safe. Upon initiation of revisional surgery after OAGB, it is recommended to assess the pouch size and volume, the length of BPL, the total small bowel length, and the CC length. The revision should be tailored to each patient, taking these factors into consideration.

Clearly, the mechanism of OAGB relies on restriction and hypo-absorption, and it is obvious that the only long-lasting effect is hypo-absorption. After the restriction wanes, the degree of hypo-absorption will define the final results of surgery. In this sense, the measurement of the total small bowel length is crucial in obtaining the desired outcomes in revisional surgery. In this cohort, during follow-up, patients without BPL elongation had the lowest weight loss outcomes. The mean CC length in this group was 455 cm, while in the other groups it was 250-280 cm. In cases of BPL elongation after OAGB, many patient factors are taken into consideration regarding the length, including age, number of previous MBS, BMI, severe obesity-related diseases, the patient’s compliance with medical treatment and surveillance, BPL length, and total small bowel length. There is no precise target, but a more tailored approach is selected. The conversion of OAGB to RYGB without BPL elongation is probably not effective in weight loss terms. Sargsyan et al. [24] reported in their systematic review and meta-analysis that patients converted to RYGB have weight regain during the follow-up and noted that patients undergoing conversion to RYGB should be counseled that conversion to RYGB will not provide any weight loss.

The limb lengths selected in some patients in our cohort are not what is routinely recommended by other studies, and this could potentially increase the complication rate in these patients. In a review by Eagleston and Nimeri [25] regarding the optimal limb lengths in RYGB, they note that when performing distalization for WR after RYGB, a CC of at least 200 cm and a total alimentary limb of at least 400 cm is necessary to minimize the risk of protein-calorie malnutrition. The revisional options for patients with unsatisfactory weight loss after OAGB are not clearly defined. These patients are at increased risk for both short- and long-term complications and, in addition, at more risk for an additional failure (IWL/WR). When we approach such a patient, a comprehensive discussion is held with the patient, and a shared decision is then taken regarding the revisional surgery and its hypo-absorptive intensity, weighing the benefits and the potential risks. Following this, in some cases a more “radical” option is chosen and shorter limb lengths are structured. In this relatively small cohort, and during the relatively short 19-month follow-up, there were no serious nutritional issues, and only one patient had mild iron deficiency.

There are several limitations to this study; it is composed of a relatively small number of patients, with no control group, and clear conclusions could not be drawn. In addition, it is retrospective in nature and represents the experience of a single surgeon. Despite these limitations, to our knowledge, this is the first study reporting various techniques of surgical revisions due to WR/IWL after OAGB. Further larger cohort studies should be conducted to clarify what is the most effective revisional procedure for WR/IWL after OAGB.

## Conclusions

In conclusion, WR/IWL after OAGB can be encountered during follow-up. The reason for WR/IWL after OAGB varies, and surgical revision should be tailored to each patient. In this case series, several options for surgical revisions were performed. It was shown that there is a relatively high safety profile both in the short and long term. In terms of efficacy, BPL elongation, conversion to RYGB with BPL elongation, or conversion to SADI-S correlated with better weight loss outcomes. The scientific literature lacks sufficient data on the surgical management of WR or IWL following OAGB. We call more centers to publish their experience to help identify methods that yield favorable outcomes.
